# Supplementary material for: Exploring the genetic architecture underlying dietary fiber content in Colombian Andean blueberry (Vaccinium meridionale Swartz)
Source: PLoS One. 2026 Jun 4;21(6):e0344321. doi: 10.1371/journal.pone.0344321 (PMC13235929; doi:10.1371/journal.pone.0344321)

**S1 Fig.** **Spearman´s correlation coefficients** including IDF: Insoluble dietary fiber; SDF: Soluble dietary fiber; TDF: Total dietary fiber; SDF/IDF x 100 ratio, the altitude where each genotype was sampled and the maturity index. * denotates *p* < 0.05; ** denotates *p* < 0.01; *** denotates *p* < 0.001.


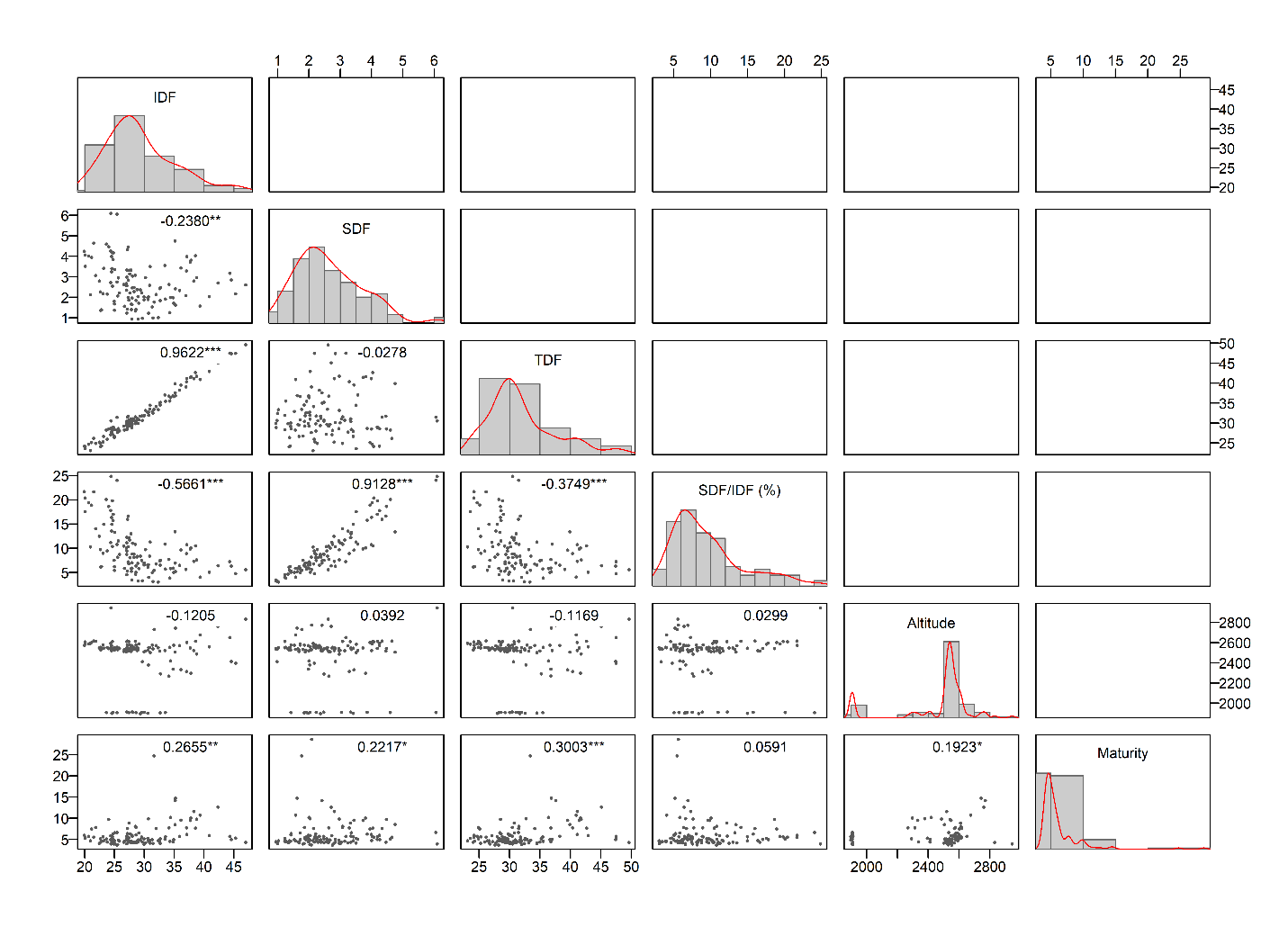

Supplement: S1 Fig — * Denotates p < 0.05; ** denotates p < 0.01; *** denotates p < 0.001. (DOCX) [file pone.0344321.s004.docx]
