# Supplementary material for: Exploring the genetic architecture underlying dietary fiber content in Colombian Andean blueberry (Vaccinium meridionale Swartz)
Source: PLoS One. 2026 Jun 4;21(6):e0344321. doi: 10.1371/journal.pone.0344321 (PMC13235929; doi:10.1371/journal.pone.0344321)

**S2 Fig.** **Box-plot displayed for each of the measured response variables in the fruit collection**, including the control commercial fruit. IDF: Insoluble dietary fiber; SDF: Soluble dietary fiber; TDF: Total dietary fiber; and SDF/IDF x 100 ratio.


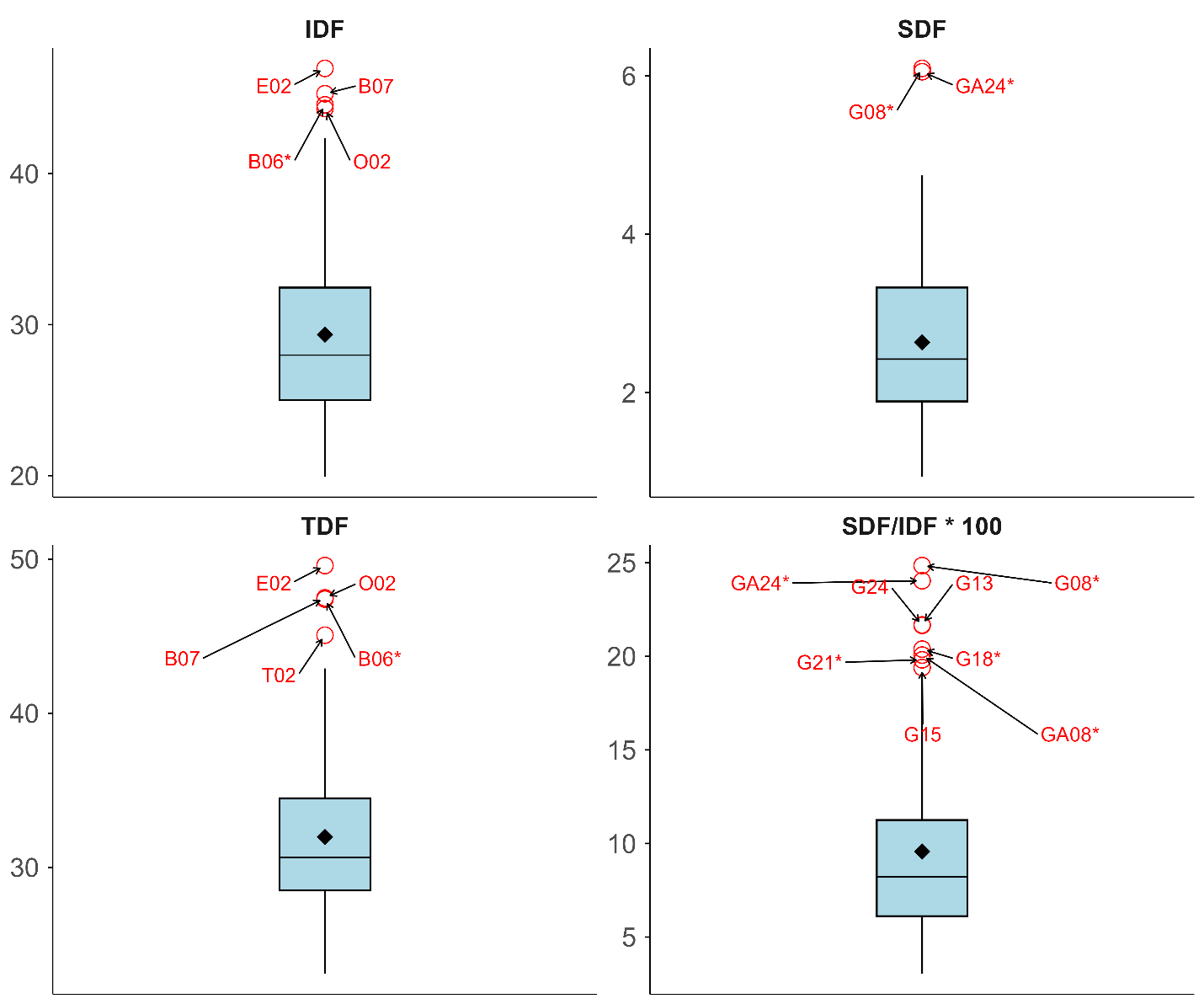

Supplement: S2 Fig — IDF: Insoluble dietary fiber; SDF: Soluble dietary fiber; TDF: Total dietary fiber; and SDF/IDF x 100 ratio. (DOCX) [file pone.0344321.s005.docx]
