# Supplementary material for: Exploring the genetic architecture underlying dietary fiber content in Colombian Andean blueberry (Vaccinium meridionale Swartz)
Source: PLoS One. 2026 Jun 4;21(6):e0344321. doi: 10.1371/journal.pone.0344321 (PMC13235929; doi:10.1371/journal.pone.0344321)

**S3 Fig.** Population structure analysis of the *V. meridionale* genotypes. LEA admixture coefficient analysis, K1 (green), K2 (purple) and K3 (orange).


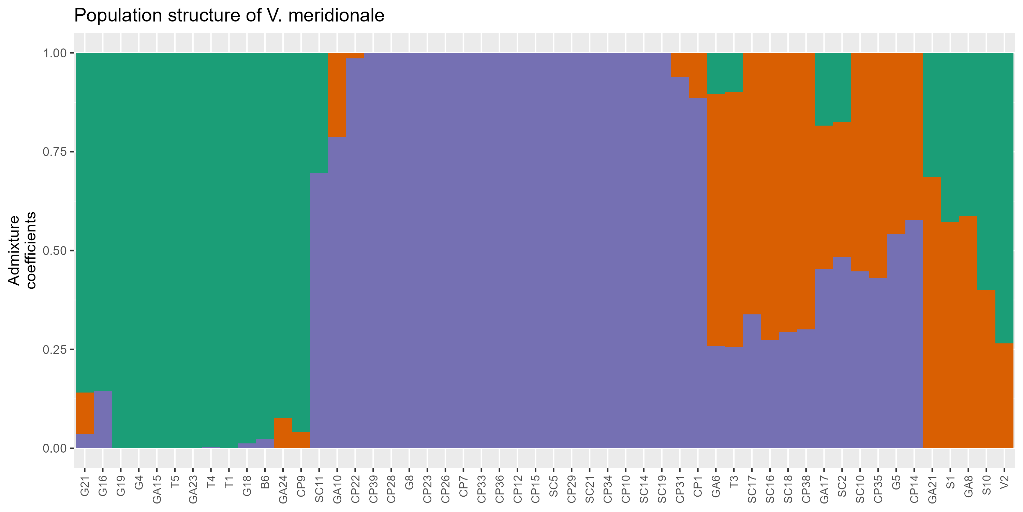

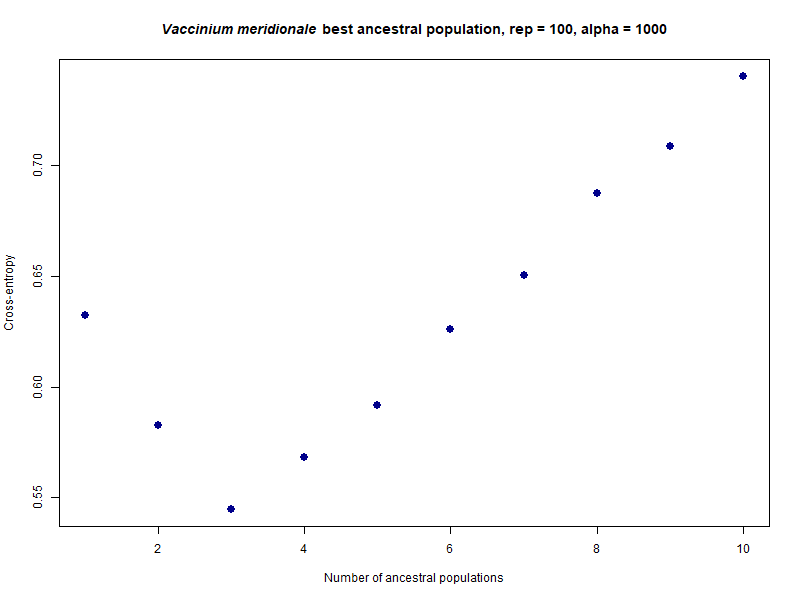

Supplement: S3 Fig — LEA admixture coefficient analysis, K1 (green), K2 (purple) and K3 (orange). (DOCX) [file pone.0344321.s006.docx]
