# Supplementary material for: Exploring the genetic architecture underlying dietary fiber content in Colombian Andean blueberry (Vaccinium meridionale Swartz)
Source: PLoS One. 2026 Jun 4;21(6):e0344321. doi: 10.1371/journal.pone.0344321 (PMC13235929; doi:10.1371/journal.pone.0344321)
Supplement: S4 Fig — (DOCX) [file pone.0344321.s007.docx]

**S4 Fig.** Linkage disequilibrium (LD) decay at the genomic level calculated for the *V. meridionale* panel.

**
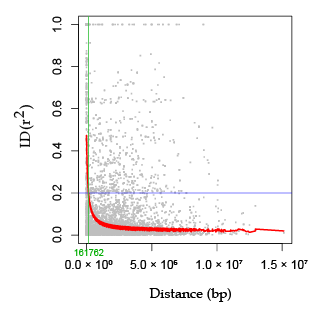
**
